# Facts and controversies regarding oral health in Parkinson's disease: A case-control study in Spanish patients

**DOI:** 10.4317/medoral.25348

**Published:** 2022-04-03

**Authors:** Ana María García-De-La-Fuente, Irene Lafuente-Ibáñez-de-Mendoza, María José Lartitegui-Sebastián, Xabier Marichalar-Mendia, María Ángeles Echebarria-Goikouria, José Manuel Aguirre-Urizar

**Affiliations:** 1 Department of Stomatology II, Faculty of Medicine and Nursery, University of the Basque Country (EHU). Leioa. Spain; 2 Department of Nursing I, Faculty of Medicine and Nursery, University of the Basque Country (EHU). Leioa. Spain

## Abstract

**Background:**

Parkinson's disease (PD) is one of the leading neurological disorders, affecting more than 6 million people worldwide. These patients present motor and non-motor symptoms, including oral pathology. The objective of this research is to determine the oral health of patients diagnosed with PD, in order to stablish a specific preventive oral health programme.

**Material and Methods:**

Case-control study on 104 PD and 106 control patients. The pre-designed clinical protocol included a complete oral examination on general aspects, standardised epidemiological index for caries, periodontal disease and edentulism, analysis of oral hygiene, presence of mucous/ salivary/ functional disorder, and dental treatments.

**Results:**

A higher number of PD patients consumed daily sweets (*p*<0.004) and antidepressant drugs (*p*<0.004). Patients with PD practised less interdental hygiene (*p*<0.023). The mean plaque index was higher in PD (*p*<0.003). Drooling (*p*<0.001), xerostomia (*p*<0.001), hyposialia (*p*<0.001), dysphagia (*p*<0.001), hypogeusia/dysgeusia (*p*<0.025) and chewing difficulty (*p*<0.006) were more common in PD.

**Conclusions:**

Oral disorders are frequent in PD. A good knowledge of these alterations will allow us design a specific preventive protocol. Some oral alterations may be a sign of diagnostic alert or progression of PD.

** Key words:**Parkinson's disease, oral health, dysgeusia, dysphagia, drooling.

## Introduction

Neurological diseases are the second leading cause of human mortality (1). Parkinson's disease (PD) is the most prevalent neurological disorder after Alzheimer's disease and affects more than 6 million people (1). It is estimated that 0.3% of the general population suffers from PD, reaching 3% in men over 80 (2).

In PD there is a progressive degeneration of dopaminergic neurons in the substantia nigra that leads to motor impairment, causing tremor, rigidity, bradykinesia, postural instability, dysphagia, etc. These patients also present non-motor symptoms including cognitive impairment and autonomic and psychiatric disorders (2). The oral pathology of patients with PD has previously been analysed but with limited and, in many cases, controversial results, due to poor methodological design such as absence of a control group, little collection of data with questionnaires and lack of professional oral examination (3,4). To date, some cross-sectional case-control studies have been conducted, but none on Spanish PD patients.

With this background, we designed the current study to analyse the oral health status of patients diagnosed with PD in the Basque Country (Spain), in order to stablish a specific preventive oral health programme.

## Material and Methods

- Study population

A cross-sectional case-control study was conducted on 104 patients diagnosed with PD, Parkinson's disease group (PDG), which corresponded to 66 men and 38 women with a mean age of 66.19+9.3 years; and 106 healthy control patients, control group (CG), that consisted of 37 men and 69 women with a mean age of 59.26+14.11 years.

The study was performed at the Dental Clinic Service of the University of the Basque Country/ EHU in collaboration with ASPARBI (Parkinson's Association of Bizkaia). All patients were examined at the same time, between 15:00 and 19:00 hours.

Inclusion criteria for PDG cases were: 1) Having a confirmed diagnosis of Parkinson's disease by a Neurology Service, and 2) Being over 18 years of age at the time of the study. Inclusion criteria for the CG controls were: 1) Not being diagnosed with Parkinson's disease or any other neurological disease, and 2) Being over 18 years of age at the time of the study.

- Parameters assessed

A specific protocol was designed for the extraction of data, in which general information, oral hygiene habits, nutritional aspects, presence and characteristics of prosthetic and restorative treatments and dental/ periodontal/ mucous and salivary gland disorders were collected, as well as oral functional alterations, pharmacological therapies and presence of other systemic diseases. The clinical protocol included a complete oral examination, in order to evaluate the standardised epidemiological index for caries, periodontal disease and edentulism, analysis of oral hygiene, presence of mucous, salivary and functional disorders, and dental treatments.

In all cases a complete anamnesis and orofacial examination was performed, in a conventional dental chair, under artificial light, using oral mirrors, Wilkings-Tufts Explorer 17-23 (Explorer WT 17-23 Silver, Hu-Friedy, Mfg. Co. LLC, Chicago, USA) and a standardised periodontal probe (PCP-11, Hu-Friedy, Mfg. Co. LLC, Chicago, USA). The exploration protocol assessed the following index and data: 1) Decay-missing-filled index (DMF) index, 2) Community Periodontal Index of Treatment Needs (CPITN), 3) Plaque Index (PI), 4) Clinical attachment loss (CAL) (Mild: 1-2 mm / Moderate: 3-4 mm / Severe: 5 mm), and 5) Dichotomous index for the presence of peri-implant disease. The presence of hyposialia was assessed by indirect clinical signs (5).

Prior to the study, sessions were held to homogenise the assessment criteria among the different clinicians (ILIM, AMGF, MJLS, MAEG and JMAU), in order to achieve maximum concordance. All patients were assessed by at least two assessors. Discrepancies were resolved by consensus and in cases where this was not possible, a third assessor (AMGF, JMAU) was always involved.

- Statistical analysis

A univariate description was performed, describing the quantitative (mean and standard deviation) and qualitative variables (frequency and percentage). For the analysis, Pearson Chi-square test was used when the two variables were qualitative, and Student's t-test when one variable was quantitative and the other qualitative. It was considered statistically significant when *p*<0.05. The statistical analysis was performed with IBM SPSS v.23 software.

## Results

- General data

Data on the general characteristics of both groups, as well as nutritional habits, oral hygiene, drugs they received and presence of other systemic diseases are shown in Table 1.

The PDG had an older mean age and a higher number of men than the CG (*p*<0.001). The percentage of smokers was significantly lower in patients with PD (*p*<0.001), although alcohol consumption was similar. The daily intake of sweets was more frequent in the PDG group (*p*<0.004).

The majority of patients in both groups reported brushing their teeth more than twice a day. However, those in the CG group used interdental hygiene procedures more commonly than those in the PDG group (*p*< 0.023).

**Table 1:**
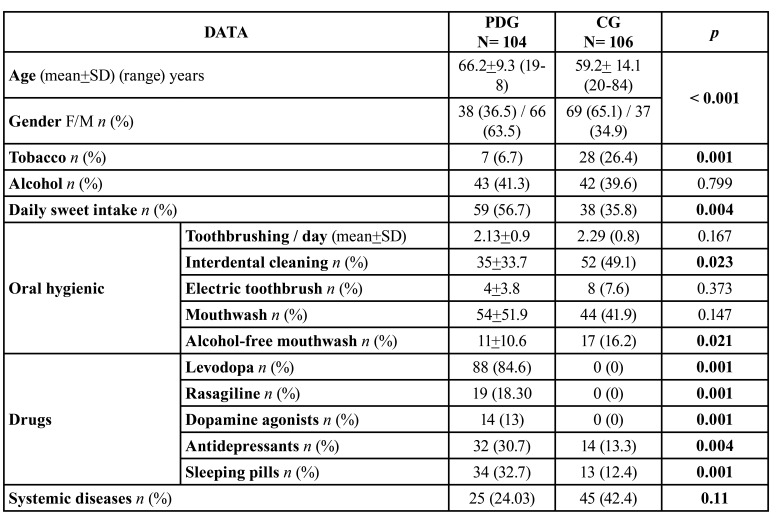
Main data of the study groups. (PDG and CG).

PD patients were less likely to use both interdental brushing (PDG: 19.2% vs CG:29.30%) and flossing (PDG: 10.6% vs CG:16%). About 50% of the patients in both study groups supplemented their oral hygiene with daily use of mouthwashes, mainly alcoholic (*p*<0.021).

Levodopa was the most frequently used medication by patients with PD, alone or in combination with ragasiline and/or dopamine agonists. Taking antidepressants was significantly more common in the PDG group (*p*<0.004), as well as sleeping pills, mainly benzodiazepines (*p*<0.001).

The most frequent systemic disease in both groups was arterial hypertension (PDG: 12.5% vs CG: 19.8%), followed by cardiovascular pathology (PDG: 7.7% vs CG: 2.8%), and rheumatological diseases (PDG: 4.8% vs CG: 5.7%). The majority of PDG patients (16/25) had more than one additional systemic disease.

- Dental pathology and treatments

Data on the dental pathology and treatments of both subgroups are shown in Table 2.

PDG patients had a higher mean DMF index and a greater number of teeth with active caries than CG patients. The number of missing teeth was also similar in both study groups, with only 2 patients in the PDG group and 5 in the CG group having total edentulism. As expected, there was a higher number of missing teeth and CAO index in patients older than 65 years in both study groups.

Both the CPITN index and the percentage of patients with periodontitis were higher among patients with PD, without being significant. In relation to CAL, number of patients with moderate or advanced attachment loss ( 3 mm) was significantly higher in the PDG group (*p*=0.027), as well as mean plaque index (*p*=0.003) (Table 2), which again was significantly lower amongst women in the PDG group (PDG:66 22.1 vs CG: 60.32 25.56) (*p*=0.033).

The percentage of patients with fixed prostheses was similar, but that with removable prostheses was slightly higher in the PDG group. The overall number of complete removable prostheses was low in both groups (PDG: 8.7% vs CG: 7.6%) (Table 2). In the PDG group, fixed restorations on natural teeth were more frequent (43.4%) than on implants (5.7%). Restorative treatments on dental implants in the CG group doubled that of the PDG.

- Mucous and salivary disorders

The mucous disorders in the two subgroups are shown in Table 3, and these were recognised in 33.6% of patients in the PDG group, compared to 20.7% of CG group. Tongue disorders and prosthesis-related disorders were the most common in the PDG group, with no significant differences between the CG group.

**Table 2:**
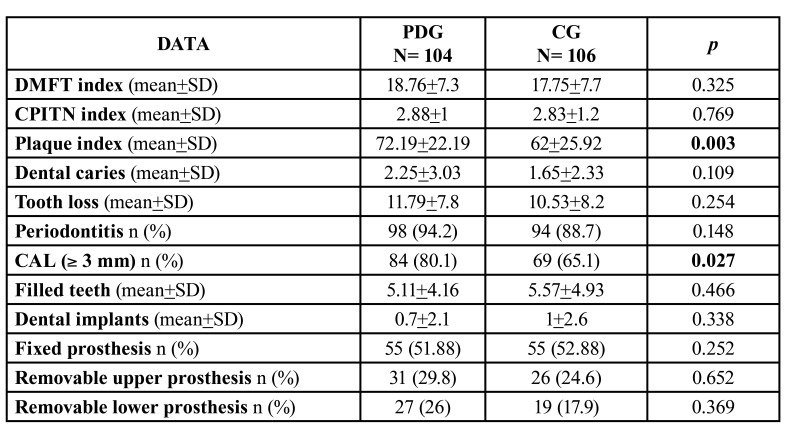
Dental pathology, periodontal pathology and dental treatments of the study groups.

**Table 3:**
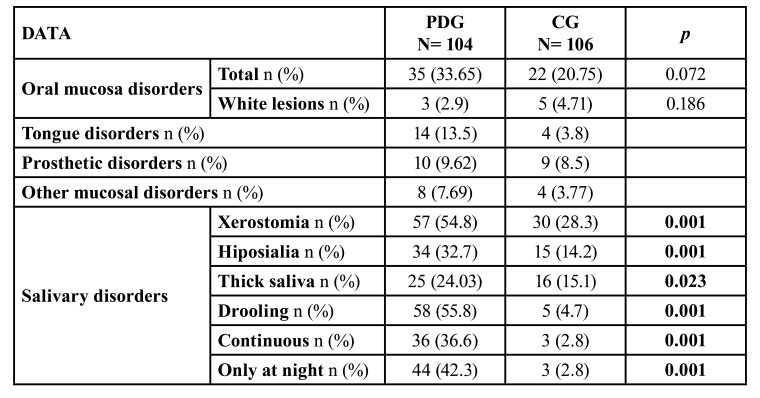
Oral mucosa pathology and salivary gland pathology of the study groups.

On oral examination, there was a significantly higher percentage of patients suffering from drooling (*p*<0.001), xerostomia (*p*<0.001), hyposialia (*p*<0.001) and thick-mucoid saliva (*p*<0.023) in the PDG group. Similarly, we identified a significant association between hyposialia and the use of antidepressant drugs (*p*<0.022) in patients with PD. More than half (n: 53) of the PDG patients had drooling and reported xerostomia.

- Functional disorders

Oral function disorders are shown in Table 4. Dysphagia was significantly more frequent in patients of the PDG group (*p*<0.001), mostly for solid food intake (*p*<0.001). The presence of taste alterations (hypogeusia and/or dysgeusia) was also significantly more common in patients with PD (*p*<0.025). Similarly, the percentage of patients who had problems with correct mastication was twice as high in the PDG group than in the CG group (*p*<0.006).

We recognised that 31% (n: 30) of the patients with PD had drooling and dysphagia and 26.3% (n=26) xerostomia and dysphagia, without these associations being significantly different in contrast to the CG group. However, we did observe a statistically significant link between drooling and dysphagia (46.7%) in women with PD (*p*<0.03).

**Table 4:**
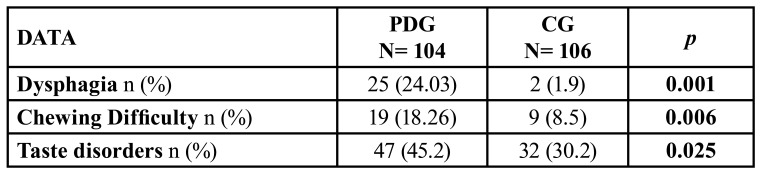
Oral function disorders of the study groups.

## Discussion

Parkinson disease is an important and frequent chronic progressive neurodegenerative pathology, in which different oral disorders can affect the overall health of patients and jeopardize their quality of life, self-esteem and socialisation (3,6).

The oral health of these patients has been previously analysed but the results are heterogeneous (3,7). There is not a consensus on the most prevalent orofacial pathologies in patients with PD (4). Therefore, a thorough discussion, focussing only on the studies that performed a complete oral clinical examination, is needed (6,8-20).

Our study reassures that men over 60 suffer more frequently from PD (9,12,13,17,19). This result partially explains why women in our study had a lower mean age in the CG group, which could be related to the recruitment of controls, and to the fact that in Spain more women demand of oral care than men (21).

Only a few patients in the PDG group smoked at the time of the study, unlike those in the CG group, where the number tobacco consumers were higher, similar to the general Spanish population. A lower risk of developing PD has been observed in smokers, indicating that smoking could have a protective effect against PD (22). On the other hand, a higher frequency of non-alcohol consumers has been described in PD patients (23); although daily consumption of alcohol was similar in the two subgroups. We did not identify any association between oral health features and drug intake, except for antidepressants, whose intake was significantly higher in the PDG group and, as expected, strongly linked to hiposialia (24).

An increased intake of sweets has been related to ageing, PD and other psychiatric disorders like depression, probably as a reward mechanism associated with dopamine (6,7). Depression is a common complication of PD, that we also recognised in our study (25). Therefore, an excessive intake of sweets in a PD patients should be an important feature to take into account for suspicion of occult depression (7).

It is known that patients with PD have a significantly higher intake of sugar and sweets (26). Although this data may be linked to a greater cariogenic pathology, dental caries numbers in the PDG and CG group were similar, and lower than in previous studies (9,10,13,17,20). These findings could be related to the good oral hygiene in our patients, who mostly cleaned their teeth more than twice a day. However, the significantly higher mean plaque index in the PDG group pointed a frequent but defective oral hygiene (10,13).

This increased plaque accumulation in patients with PD has been associated with motor difficulties in brushing, that worsen as the degenerative disease progresses (8,15,18,19). In our cohort, a small number of patients with PD used an electric and interdental toothbrush, which would facilitate and improve their procedure (13,17,19). This incapacity for fine motor skills like performing good oral hygiene, leads to development of caries, periodontitis and tooth loss. Therefore, this inability must be important data to check for dentists, as it may be a diagnostic warning sign of PD, or its aggravation (19).

We observed a high mean DMF index in PD patients, but less active caries in PD patients (9,10,13,17,18,20). These findings do not confirm the link between untreated caries and progression of PD (14). Likewise, we could not identify a direct relationship between caries and high daily consumption of sweets (9). The increased dental care and improved oral hygiene of our patients (21), may explain why the number of missing teeth was slightly lower in the PDG group than in previous studies (8,15,17,20).

We were not able to identify any specific oral mucosal disorder in patients with PD, but these were more common in the PDG group, contrary to what was previously reported (6). The presence of periodontitis was very high in both groups in our study (6), but different from other studies (12,13,). These findings match with the Spanish population (21). The significantly higher moderate-advanced CAL in the PDG group could be explained by the difficulty of patients for interdental plaque removal. Similar to that observed by Ribeiro *et al*. (20), most of PD patients had fixed crowns and bridges, and less removable complete dentures.

Salivary alterations are considered a special aspect in the clinical spectrum of PD. Xerostomia, with or without hyposialia, was significantly more frequent in our PDG group (6,10,15). The symptomatic perception of dry mouth (xerostomia) was higher than the existence of hyposialia in PD patients. Almost a quarter of these patients had thick-mucoid saliva. We think this finding may be linked to the autonomic innervation alteration of the major salivary glands, leading to a lower serous salivary secretion.

Diurnal, nocturnal (pre-droolers), or all-day drooling is a classic and underestimated salivary clinical sign of PD, whose onset classically occurs in the early stages of PD, and nocturnal nature is often overlooked and assumed to be inherent to the disease (6). This progressive disorder is an important clinical feature that significantly affects the quality of life of patients (6,11). and can lead to respiratory complications. Overall drooling and nocturnal drooling were more common than previous studies (27). The pathogenesis of this disorder in PD is multifactorial, but mainly related to a decreased frequency of automatic swallowing, anterior flexed head position, oropharyngeal bradykinesia and dysphagia (10,11,27). Moreover, PD patients do not have hypersialorrhoea per se, but rather an alteration in saliva swallowing that causes salivary accumulation and drooling. We could not find any association between drooling and dysphagia, except in the subgroup of women with PD. The frequent appearance of dysphagia (80% of patients with PD) highlights its role as a progressive motor nerve impairment indicator (28). PD patients also reported a greater difficulty in performing regular chewing (4), probably due to motor impairment.

We found that patients in the PDG presented significantly more taste disturbances than those in the CG, both in terms of bad or no taste at all. Although previous reported numbers of have been lower (29), dysgeusia could be an important warning symptom to analyse in adult males, since it may be the beginning of PD and altered sense of taste is one of the highest complaints in early PD diseases (4).

In summary, this clinical study highlights the importance of oral disorders in PD. Dentists should be aware of these progressive motor impairments related oral disorders, in order to stablish specific preventive protocols that improve the oral hygiene of these patients (use of electric toothbrushes, interdental brushes, etc.), control the intake of sweets and enhance the use of antidepressant agents. We did not observe any specific oral, salivary or functional disorder in PD patients. Some oral disorders such as dysgeusia, dysphagia and drooling may constitute a diagnostic or progressive warning sign of PD.
